# Identification of a Predictive Biomarker for the Beneficial Effect of Keishibukuryogan, a Kampo (Japanese Traditional) Medicine, on Patients with Climacteric Syndrome

**DOI:** 10.1155/2014/962109

**Published:** 2014-01-20

**Authors:** Takao Namiki, Hiromi Sato, Yukari Matsumoto, Haruka Kakikura, Koichi Ueno, Atsushi Chino, Hideki Okamoto, Akito Hisanaga, Akiyo Kaneko, Toshiaki Kita, Maki Kihara, Makio Shozu, Katsutoshi Terasawa

**Affiliations:** ^1^Department of Japanese-Oriental (Kampo) Medicine, Graduate School of Medicine, Chiba University, Chiba 260-8670, Japan; ^2^Department of Geriatric Pharmacology and Therapeutics, Graduate School of Pharmaceutical Sciences, Chiba University, Chiba 260-8670, Japan; ^3^Center for Preventive Medical Science, Chiba University, Chiba 260-0856, Japan; ^4^Chiba Chuo Medical Hospital, Chiba 264-0017, Japan; ^5^Department of Reproductive Medicine, Graduate School of Medicine, Chiba University, Chiba 260-8670, Japan

## Abstract

*Keishibukuryogan* (KBG; *Guizhi-Fuling-Wan* in Chinese) is one of the Kampo (Japanese traditional) medicines used to treat patients with climacteric syndrome. KBG can be used by patients who cannot undergo hormone replacement therapy due to a history of breast cancer. We evaluated whether cytosine-adenine (CA) repeat polymorphism of the estrogen receptor **β** gene can be a predictor of the beneficial effect of KBG on climacteric syndrome. We also investigated the relationship between CA repeat polymorphism, the patients' profiles, and the therapeutic effect. We found that CA was an SS, SL, or LL genotype according to the number of repeats. We studied 39 consecutive patients with climacteric disorders who took KBG for 12 weeks. The diagnosis of climacteric disorders was made on the basis of the Kupperman index. KBG significantly improved the patients' climacteric symptoms (i.e., vasomotor symptoms in the patients with the LL genotype and melancholia in the patients with the SL genotype). No relationship between the patients' profiles and CA repeat polymorphism was recognized. CA repeat polymorphism could thus be a potential biomarker to predict the efficacy of KBG in climacteric syndrome, and its use will help to reduce the cost of treating this syndrome by focusing the administration of KBG on those most likely to benefit from it.

## 1. Introduction

Climacteric syndrome, which is caused by a decrease of the estrogen level during menopause, often severely impairs a woman's quality of life [1, 2]. Many climacteric women suffer from vasomotor symptoms, mood disorders, vaginal dryness, headache, shoulder stiffness, and other problems. Hormone replacement therapy (HRT) is one of the most effective treatments for climacteric symptoms, especially for vasomotor symptoms, mood problems, and vaginal dryness. In recent years, however, epidemiological studies have reported adverse reactions to HRT such as an increase in the risks for stroke, deep vein thrombosis, dementia, and breast cancer [3–6]. In Japan, the use of Kampo medications (Japanese herbal medicine) is one alternative for controlling these symptoms in women who reject HRT or who cannot receive HRT due to a history of breast cancer [7].

*Keishibukuryogan* (KBG; *Guizhi-Fuling-Wan * in Chinese) is a Kampo medicine that has been effective in patients with climacteric syndrome. KBG consists of five crude drugs: cinnamon bark (*Cinnamomum cassia* Blume), peony root (*Peonia lactiflora* Palls), peach kernel (*Prunus persica* Batsch), poria sclerotium (*Poria cocos* Wolf), and moutan bark (*Peonia suffruticosa *Andrews). However, at present, the clinical evidence of KBG's efficacy is still limited because there have been few large-scale clinical trials. In addition, in a recent randomized study in the United States, patients with hot flashes did not show improvement as a result of KBG treatment, suggesting that the inclusion criteria of patients in clinical trials are important in showing the efficacy of Kampo medicine [8].

In 1996, the estrogen receptor (ER) *β* gene was identified in an animal study [9]. Five isoforms of ER*β* were subsequently confirmed in human ovarian tissue [10]. ER*β* expression has also been observed in all types of granulose cells of the follicle [11], and polymorphisms of the ER*β* gene are associated with abnormal ovulation [12]. The ER*β* gene is suggested to play an important role in ovarian function. A retrospective study by our group revealed that the cytosine-adenine (CA) repeat polymorphism of the ER*β* gene is correlated with climacteric symptoms [13]. In the present prospective study, therefore, we aimed to clarify the association between the CA repeat polymorphism of the ER*β* gene and the therapeutic effects of KBG on women with climacteric symptoms. We evaluated whether the contribution of the CA repeat polymorphism of the ER*β* gene has a role in the effectiveness of KBG in women with climacteric syndrome.

## 2. Subjects and Methods

### 2.1. Subjects

The subjects were 39 consecutive females over 40 years old with climacteric symptoms at Chiba University Hospital and Chiba University's Kashiwanoha Clinic. Of these, 18 subjects were postmenopausal and the others were not yet menopausal. Menopause was diagnosed when the patient had not had a period in the past 12 months. The inclusion criterion of climacteric syndrome was a Kupperman index (KI) score over 20. The KI represents the total score of 11 components including vasomotor symptoms (hot flashes), paresthesia, insomnia, nervousness, melancholia, vertigo, concentration disorders, arthralgia or myalgia, headache, palpitations, and formication (a sensation of insects crawling over the skin). We simultaneously defined menopause via blood measurement as a follicle-stimulating hormone (FSH) level ≥40 mIU/mL, as an estradiol (E_2_) level ≤20 pg/mL, or as an anti-Müllerian hormone (AMH) of <14.3 pmol/L as a reference of ovarian reserve. We excluded patients with physical diseases such as thyroid disease via blood examinations and psychiatric diseases such as depression via Self-rating Depression Scale (SDS) questionnaires.

### 2.2. Study Objective and Design

We investigated (1) the relationship between the CA repeat polymorphism and the KI before treatment with KBG and (2) the efficacy of KBG by comparing the total or component scores of the KI before and after treatment with KBG.

All patients took KBG extract granules at a dose of 2.5 grams (TJ-25, Tsumura Co., Tokyo) three times a day for 12 weeks. During the study, the patients came to our outpatient clinic every 4 weeks, and they underwent blood tests at the first visit and the final visit after the 12 week treatment was completed. The cessation or any change in the KBG treatment for any reason (including at the request of the patient) was considered dropping out of the study.

We explained the objectives of the study to each patient by asking her to fill out questionnaires (including Kupperman index, menstrual cycle, preset symptoms, and past history questionnaires) and obtained written informed consent from all patients. The study questionnaires were completed by patient interviewers.

We analyzed the correlation of each component of the KI with the detailed climacteric symptoms. We examined all patients for the CA repeat of the ER*β* gene. The number of CA repeats was classified into two categories: ≤21, defined as short (S), and >21, defined as long (L). We defined the three genotypes involving combinations of alleles as SS, SL, and LL.

This study was approved by the ethics committees of the Graduate School of Medicine, Chiba University, the Graduate School of Pharmaceutical Sciences, Chiba University, and the Chiba University Environment Field Science Center. In addition, as genetic information and personal medical information are critical to personal privacy, we managed all data very carefully with a stand-alone personal computer.

### 2.3. Analysis of CA Polymorphisms of the ER*β* Gene

Genomic DNA was extracted from human peripheral blood leukocytes using the QIAamp DNA Mini Kit (Qiagen, Hilden, Germany) according to the manufacturer's protocol. Polymerase chain reaction (PCR) was performed in 75 *μ*L of reaction mixture with the following components: 150 ng of human genomic DNA, oligonucleotide primers designed to amplify polymorphic CA repeats in intron 6 of the human ER**β** gene (forward: 5′-CAA TTC CCA ATT CTA AGC CT-3′ and reverse: 5′-ATT CTT CTT TAG GCC AGG CA-3′) at 0.4 *μ*M, dNTP mixture (TaKaRa Bio, Otsu, Japan) at 200 *μ*M, 7.5 *μ*L of 10× reaction buffer (containing 15 mM MgSO_4_) (Transgenomic, Omaha, NE), and 2.5 U of optimase polymerase (Transgenomic). The reactions were brought to a total volume of 75 *μ*L by adding MilliQ water.

The amplification profiles were as follows: 35 cycles of denaturing at 94°C for 30 sec, annealing at 60°C for 30 sec, and extension at 72°C for 30 sec. The PCR products were purified with the QIAquick PCR Purification Kit (Qiagen) and used in the following analysis.

We conducted the analysis of the CA repeat polymorphisms by dye-terminator cycle sequencing using the Dye Terminator Cycle Sequencing Quick Start Kit (Beckman Coulter, Fullerton, CA) and the CEQ2000 DNA Analysis System (Beckman Coulter) according to the manufacturer's protocols.

### 2.4. Statistical Analysis

The statistical analysis was carried out as follows. The comparison of dispersibility between generations was done using the Bartlett test. Multiple comparisons were done using the Tukey-Kramer test in the case of equal dispersion or by the Steel-Dwass test in the case of nondispersion. The comparison of healthy subjects and patients with climacteric disorders exhibiting dispersibility was done by *F*-test and analyzed using Student's *t*-test in the case of equal dispersion or Welch's *t*-test in the case of nondispersion. The coefficient of correlation was obtained by Pearson's correlation test using PASW Statistics 18. The sensitivity, specificity, and the distribution of frequencies were calculated using cross-tabulation with PASW Statistics 18. StatLight 1997 software (Yukms, Tokyo) was used for the statistical analyses. *P* values < 0.05 were accepted as significant in all analyses.

## 3. Results 

### 3.1. Characteristics of the Subjects Enrolled in This Study

**Figure 1:**
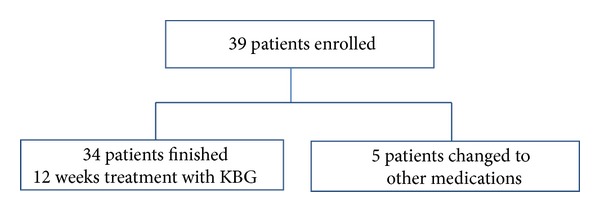
Flow chart of patients enrolled in the study.

Five of the 39 subjects dropped out; the remaining 34 patients with climacteric symptoms all took the KBG for 12-weeks (Figure 1). The postmenopause and premenopause groups were each 17 subjects. There were no significant differences between these groups except in age and height (Table 1). The past histories of 11 subjects showed myoma uterus or endometriosis. Two subjects had the history of hysterectomized. No serious adverse reaction to KBG was observed.

### 3.2. Relationship between Subjects' Profiles and CA Genotype

We classified the 34 patients into three genotypes: 9 patients as SS, 12 as SL, and 13 as LL. We compared the patients' total KI scores before treatment according to the CA genotype, and we found no significant differences in the patients' profiles among the genotypes (Table 2). There were no significant differences among the three genotype groups except for the FSH level in the LL genotype (Table 3). The high FSH and low E_2_ levels indicated that those subjects were in perimenopause.

### 3.3. Correlation between KBG's Clinical Efficacy and CA Genotypes

**Figure 2:**
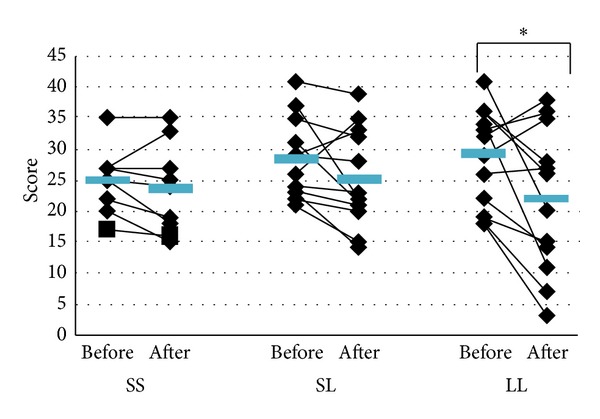
Changes in the subjects' Kupperman index (KI) from before KBG treatment to after the treatment were compared with each of the three genotypes of the CA repeat polymorphism of the ER*β* gene. Transverse bars indicate averages. *P* values (before versus after) were obtained by paired *t*-test. *P < 0.05.

To examine the effectiveness of KBG in patients with climacteric symptoms, we compared the changes in the patients' KI before and after treatment in each CA genotype. Regarding the total KI score, a significant improvement was observed only in the LL group (Figure 2). Likewise, we evaluated the improvement in the 11 climacteric symptoms included in the KI. The patients with the LL genotype showed significant improvement in climacteric KI components such as vasomotor symptoms, and patients with the SL genotype showed a significant beneficial effect on melancholia following the KBG treatment (Table 4).

## 4. Discussion

We determined the CA repeat polymorphism of the ER*β* gene in 34 climacteric patients and investigated the relationships of the CA genotypes with the patients' backgrounds and the therapeutic effects of KBG. The results suggested that KBG was effective in climacteric patients who had either the LL or SL genotype. The serum estrogen levels in the LL genotype patients, which were higher than those in the other two genotype groups, might partially explain the efficacy of KGB; although the KI values in the LL group were higher than those in the SS group.

The use of KBG for patients with climacteric syndrome can be very beneficial because KBG has no contraindications except in patients with an allergy to KBG. Because KBG has no direct effect on increasing estrogen levels, it can also be used for patients with climacteric syndrome who have a history of breast or ovarian cancer, both of which are contraindications for HRT.

In ERs, many genetic polymorphisms including single-nucleotide polymorphisms (SNPs) and a microsatellite polymorphism have been reported. In 1998, Tsukamoto et al. first characterized the CA repeat polymorphism (D14S1026) of the ER*β* gene in a Japanese population [14]. A systematic mutation screening subsequently detected five different sequence variants, including two mutations and three polymorphisms [15]. Importantly, in an in vitro functional analysis, the presence of the valine in position 320 showed significantly decreased maximal transcriptional activity [16]. The number of CA repeats ranges from 13 to 30.

The correlation of the CA repeat polymorphism with various diseases has been reported in breast cancer [17], endometrial cancer [18], osteoporosis [19], and Alzheimer's disease [20]. A relationship between the CA repeat polymorphism and the androgen concentration [21] and prolactin levels [22] has also been reported, which strongly suggests that the CA repeat polymorphism is related to the secretion of sexual hormones. The distribution of the number of repeats of CA exhibits racial differences; there is a single peak of the specific CA repeat number in Japan and China [19, 23], while a bimodal distribution has been shown in Caucasians and in India [18, 24]. The racial differences in the distribution of the CA repeat polymorphism may explain why there have been no significant results of clinical trials in the United States.

In our previous study, women with the SS genotype were shown to have an increased risk of perimenopausal symptoms and menopause-related psychological and vasomotor symptoms, and they required HRT to control their severe climacteric symptoms. It has been reported that most patients with the SS genotype show strong vasomotor symptoms, and HRT would likely be used to treat them [13, 25]. ER*β* genotypes might relate to the severity of vasomotor symptoms in climacteric syndrome. In the present study, the KBG treatment had no significant effect on vasomotor symptoms in the SS genotype group but it was effective against these symptoms in the LL genotype group. Although our LL patients had high KI scores, vasomotor symptoms were improved in the LL group. This result is related to the “Sho” diagnosis in Kampo medicine (“Sho” diagnosis is diagnostic steps to determine Kampo medicines).

The mechanism of vasomotor symptoms in patients with climacteric syndrome remains unclear. One mechanism that has been reported is the increase of the calcitonin gene-related peptide (CGRP) concentration, which has the effect of microvascular dilation [26], and another is the lowering of blood sex hormone-binding globulin (SHBG) levels due to a decreased blood estradiol concentration [27]. However, it was reported that premenopausal patients with a relatively short CA repeat of the ER*β* gene had higher serum levels of SHBG compared to those with a longer repeat region [21].

Westberg et al. reported that their patients with short CA repeat had higher SBGH levels which might be derived from higher androgen (testosterone) levels, although there was no correlation between E2 levels and genotype [21]. They also checked the relationship between prolactin levels and CA repeats, and they observed a significant difference in prolactin levels: their “SS + SL” group had significantly higher prolactin levels compared to the “LL” group, but there was no such correlation for estradiol and prolactin in any of the subgroups with respect to ER*β* [22]. It remains unknown whether ER*β* polymorphism has any effect on the SHBG level.

The release of hormones may be affected by the menstrual period. Westberg et al. [21] noted that serum samples are obtained in the follicular phase when the levels of estradiol are relatively low, although doing so is difficult in clinical practice. However, Comings proposed that even though the CA repeat region in the ER*β* is situated in a nontranslated region, there are several mechanisms that might explain such a relationship between short repeats in nontranslated regions and gene function, including their potential to form alternative DNA structures (such as Z-DNA) that modulate transcriptional activity [28].

Regarding the mechanism of KBG as a treatment for vasomotor symptoms, it has been reported that the increase in skin temperature is inhibited by the normalization of the high estrogen-independent sensitivity of the CGRP receptor [29, 30]. KBG shows estrogen-like activity [31]. In addition, because KBG shows an agonistic activity for ER*β*, KBG has been suggested to act through ER*β* [32]. In order to clarify the relationship between CGRP and vasomotor symptoms, measurements of the CGRF level are needed to determine the concentration of CGRF in each genotype. However, it is difficult to detect the peak concentration of CGRP because it occurs during vasomotor symptoms [33, 34].

In the melancholia related to climacteric syndrome, a significant improvement was shown in only the SL genotype patients in the present study. However, the trend in the KI melancholia score before and after KBG treatment was almost the same in all three genotype groups. Kanda et al. reported that Kampo medication can be more effective for the melancholia related to climacteric syndrome than HRT [35]. They reported that melancholia improved in 58% of the patients treated with Kampo medication (KBG was administrated on 25% of the patients) whereas in 33.3% of the patients treated with HRT. Kampo medication might have different effects with HRT on patients with climacteric syndrome.

In Japan, more than 80% of medical doctors prescribe Kampo medicine [7, 36]. Kampo formulae are regulated as prescription pharmaceutical drugs by the Japanese Ministry of Health and have been covered by the national health insurance for about 30 years. By using the theory of Kampo medicine, Kampo specialists analyze a patient's constitution and various complaints and then prescribe various types of Kampo medicine. Making a Kampo diagnosis is relatively difficult for nonspecialist doctors, and thus treating climacteric patients with Kampo medicines is also difficult. Analyzing the CA genotype may therefore help Kampo expert doctors as well as nonexpert doctors to choose an appropriate Kampo prescription.

With these results, we have taken the first step in using the ER*β* genotype as a predictive biomarker for the beneficial effects of KBG on patients with climacteric syndrome. A major limitation of our study was its small sample size (*n* = 39). The sample sizes for each of the three genotypes (SS, SL, and LL) were necessarily even smaller, and this may have affected our ability to determine significant differences between groups. Nevertheless, our study indicates that the ER*β* genotype can be expected to eventually be a useful biomarker as a predictor of the efficacy of KBG. Evidence relevant to the CA polymorphism should be collected through larger-scale and more detailed studies.

## Figures and Tables

**Table 1:** Clinical characteristics of the post- and premenopause patients.

	All	Postmenopause	Premenopause	*P*
	*n* = 34	*n* = 17	*n* = 17	
Age	49.5 ± 4.69	51.2 ± 5.45	47.7 ± 3.02	0.028*
Height (cm)	156.8 ± 3.46	155.6 ± 3.44	158.1 ± 3.09	0.033*
Body weight (kg)	52.7 ± 6.67	53.7 ± 7.49	51.7 ± 5.78	0.394
BMI (kg/cm^2^)	21.5 ± 3.22	22.3 ± 3.75	20.7 ± 2.44	0.278
Age of menarche	12.5 ± 1.13	12.2 ± 0.81	12.8 ± 1.35	0.121
SDS	46.2 ± 8.51	45.2 ± 9.15	47.2 ± 7.98	0.502
Hysterectomized	2	2	0	
Ratio of menopause (%) (delited hysterectomized)	44.1	88.2	0	

Data are mean ± SD. BMI: body mass index, SDS: self-rating depression scale. The *P* values (post versus pre) were obtained by Student's *t*-test or Mann-Whitney test. *P < 0.05.

**Table 2:** Clinical characteristics of 34 patients who finished the KBG treatment, classified by their CA repeat polymorphism of the ER*β* gene.

	SS	SL	LL	*P*
	*n* = 9	*n* = 12	*n* = 13	
Age	50.1 ± 2.37	51.2 ± 5.29	47.5 ± 4.86	0.100
Height (cm)	157.1 ± 3.78	155.6 ± 3.22	157.7 ± 3.39	0.502
Body weight (kg)	54.0 ± 8.26	53.3 ± 6.26	51.2 ± 6.07	0.913
BMI (kg/cm^2^)	22.0 ± 4.18	22.1 ± 3.21	20.6 ± 2.43	0.747
Age of menarche	12.6 ± 1.33	12.3 ± 1.07	12.5 ± 1.13	0.901
SDS	46.8 ± 10.29	46.0 ± 9.05	45.9 ± 7.30	0.971
KI	25.0 ± 5.12	28.4 ± 6.49	29.2 ± 7.85	0.341
Hysterectomized	0	1	1	
Ratio of menopause (%) (delited hysterectomized)	33.3	66.7	30.8	

Data are mean ± SD. S: short allele, L: long allele, SDS: self-rating depression scale, and KI: Kupperman index. *P* values (SS versus SL versus LL) were obtained by Kruskal-Wallis test or 1-way ANOVA.

**Table 3:** The patients' hormone levels before and after treatment compared by CA repeat polymorphism of the ER*β* gene.

	SS	SL	LL	*P*
	*n* = 9	*n* = 12	*n* = 13	
E_2_(pg/mL)				
Before	69.2 ± 103.9	65.2 ± 89.5	104.6 ± 142.8	0.305
After	59.0 ± 97.3	32.0 ± 41.6	78.3 ± 100.9	0.235
FSH (mIU/mL)				
Before	49.0 ± 29.0	53.4 ± 27.2	24.1 ± 29.3	0.044^#^
After	39.1 ± 21.7	55.1 ± 29.5	25.5 ± 26.7	0.051
AMH (pmol/L)				
Before	1.33 ± 1.03	1.12 ± 1.22	6.46 ± 7.53	0.095
After	1.11 ± 1.22	0.90 ± 0.48	4.42 ± 4.83	0.107

Data are mean ± SD. S: short allele, L: long allele. *P* values (SS versus SL versus LL) were obtained by Kruskal-Wallis test or 1-way ANOVA. ^#^ *P* < 0.05.

**Table 4:** Comparison of Kupperman index components before and after 12 weeks' treatment by genotypes of the CA repeat polymorphism of the ER*β* gene.

	SS	SL	LL	*P*
	*n* = 9	*n* = 12	*n* = 13	
Vasomotor				
Before	9.78 ± 2.11	10.67 ± 1.97	10.46 ± 2.03	0.583
After	9.33 ± 2.00	10.00 ± 2.70	7.08 ± 4.05*	0.116
Paresthesia				
Before	1.33 ± 1.73	1.33 ± 1.56	2.00 ± 1.83	0.563
After	1.33 ± 1.41	1.33 ± 1.30	1.38 ± 2.06	0.904
Insomnia				
Before	3.11 ± 1.76	2.83 ± 2.17	2.77 ± 2.65	0.933
After	2.44 ± 1.67	2.33 ± 2.23	2.15 ± 2.38	0.884
Nervousness				
Before	3.11 ± 1.76	3.33 ± 1.56	3.69 ± 1.97	0.747
After	3.11 ± 2.26	3.00 ± 1.60	2.92 ± 1.93	0.973
Melancholia				
Before	1.78 ± 0.97	1.92 ± 0.90	1.69 ± 1.18	0.916
After	1.56 ± 0.88	1.25 ± 0.75*	1.38 ± 1.19	0.711
Vertigo				
Before	0.78 ± 0.67	1.00 ± 0.95	0.92 ± 0.86	0.866
After	1.00 ± 0.87	0.83 ± 0.83	0.69 ± 1.03	0.590
Concentration disorders				
Before	1.67 ± 0.87	2.08 ± 0.67	2.23 ± 1.01	0.246
After	1.56 ± 0.88	2.00 ± 0.60	1.92 ± 1.04	0.491
Arthralgia or myalgia				
Before	1.67 ± 0.87	2.00 ± 0.95	2.23 ± 1.01	0.285
After	1.56 ± 0.53	2.00 ± 0.95	1.92 ± 1.04	0.397
Headache				
Before	0.78 ± 0.83	1.75 ± 1.06	1.77 ± 1.17	0.068
After	0.67 ± 0.87	1.42 ± 1.24	1.31 ± 1.11	0.281
Palpitations				
Before	1.00 ± 1.00	0.92 ± 1.00	1.08 ± 1.12	0.947
After	0.89 ± 0.78	0.75 ± 0.62	0.69 ± 0.75	0.808
Formication				
Before	0.00 ± 0.00	0.58 ± 1.00	0.38 ± 0.87	0.177
After	0.11 ± 0.33	0.33 ± 0.49	0.54 ± 0.78	0.322

Data are mean ± SD. *P* values (SS versus SL versus LL) were obtained by Kruskal-Wallis test. *P* values (before versus after) were obtained by Signed-Wilcoxon test.*P < 0.05.
